# Impact of pollution on water quality in Algerian wetlands: a comparative assessment across multiple regions

**DOI:** 10.1007/s11356-025-37340-0

**Published:** 2026-02-13

**Authors:** Ines Houhamdi, Leila Bouaguel, Haithem Aib, Aymen Djamel Eddine Harrouz, Mouslim Bara, Jenő Nagy, Moussa Houhamdi, Herta Mária Czédli

**Affiliations:** 1 Laboratory Biology, Water and Environment (LBEE), Faculty SNV-STU, University 8 May 1945 Guelma, PO. 401, 24000 Guelma, Algeria; 2 Department of Biology, Faculty of Sciences and Nature, University Mohamed Cherif Messaadia, 41000 Souk-Ahras, Algeria; 3 Pál Juhász-Nagy Doctoral School of Biology and Environmental Sciences, Department of Hydrobiology, University of Debrecen, 4032 Debrecen, Hungary; 4 Department of Electrical Engineering, University of Kasdi Merbah Ouargla, 30000 Ouargla, Algeria; 5 Research Laboratory Applied Zoology and Animal Ecophysiology, Bejaia University, Béjaïa, Algeria; 6 HUN-REN-UD Conservation Biology Research Group, Department of Botany, University of Debrecen, 4032 Debrecen, Hungary; 7 Department of Civil Engineering, University of Debrecen, 4028 Debrecen, Hungary

**Keywords:** Wetlands, Water quality, Heavy metals, Microbiological contamination, Spatial variation, Seasonal variation, Algeria

## Abstract

Wetlands play a critical role in water purification and biodiversity maintenance, yet many are increasingly affected by anthropogenic pollution. This study provides a comparative assessment of physicochemical, microbiological, and heavy metal parameters in six Algerian wetlands spanning coastal, highland, and desert regions over a full annual cycle (November 2021–October 2022). Twenty-five indicators were measured across polluted and non-polluted sites. Polluted wetlands showed significantly higher turbidity (Z = − 3.678, *p* < 0.001), organic matter (Z = − 2.123, *p* = 0.04), and heavy metals including Cu (Z = − 4.234, *p* < 0.001), Fe (Z = − 3.123, *p* = 0.002), and Pb (Z = − 3.789, *p* < 0.001). Dissolved oxygen was consistently lower in polluted sites (Z = − 2.345, *p* = 0.02). Highland wetlands exhibited elevated nutrient loads, with nitrates (Z = − 2.789, *p* = 0.01) and ammonium (Z = − 3.123, *p* = 0.002) reflecting agricultural inputs. Microbiological contamination exceeded recommended thresholds at all polluted sites, with fecal coliforms and fecal streptococci surpassing 1000 CFU/100 ml. Seasonal analysis showed higher microbial loads in the wet season and concentration of nutrients in the dry season. Overall, the results demonstrate clear spatial and seasonal variation in water quality, with polluted sites across all regions exceeding national and WHO guideline values for nutrients, heavy metals, and microbiological indicators. These findings underscore the need for strengthened monitoring and pollution-control measures in Algerian wetlands.

## Introduction

Wetlands are among the most productive ecosystems on Earth, serving as reservoirs of aquatic biodiversity and providers of essential ecological services. They play a critical role in water purification, biodiversity conservation, and climate regulation, making them indispensable to both natural systems and human societies (Prasad et al., 2002). Since wetlands provide vital services such as water purification, biodiversity support, and climate regulation (Jeethu and Kaladevi, 2025; De Groot et al. 2018; Janse et al. 2019; Mitsch et al. 2015; Palafox–Juárez et al. 2025; Yan et al. 2023), they are gaining more and more attention as they make numerous contributions to a healthy environment (Mitsch and Gossilink 2000). These ecosystems serve organic filters, water quality improver and trapper by capturing pollutants (Kennedy and Mayer 2002; Nyman 2011).

Wetlands are global phenomena, existing on every continent, ranging from arid deserts to mesic zones, and from high mountain ranges to coastal flatlands (Courouble et al. 2021; Kingsford et al. 2016). In North Africa, wetlands span regions from coastal areas to upland areas and dry deserts. Algeria, home to 2375 wetlands, 2056 natural and 319 artificial, contributes significantly to the preservation of these ecosystems (Hammana et al. 2024). A total of fifty of these locations are on the Ramsar priority list used globally (Benzina et al. 2022; Chedad et al. 2020). The Mediterranean Basin, known for its immense ecological, social, and economic values, is one of the most significant hotspots for biodiversity, featuring a high rate of species endemism (Adloff et al. 2015). Despite their importance, wetlands worldwide have undergone substantial decline. Global assessments report that 35–60% of wetland area has been lost since 1970, driven predominantly by land-use conversion, hydrological alteration, urban expansion, and pollution (Finlayson et al. 2018; IPBES 2019). These challenges, like urbanization and industrialization (Benkesmia et al. 2023; Benslimane et al. 2019; Demnati et al. 2017) impact their physicochemical and microbiological properties, which are impacted by mixed and geographic climates (Sidhoum et al. 2020; UNESCO 2022). Levels of physicochemical factors offer essential evidence about the physical and chemical characteristics of wetland waters, influencing biological processes and aquatic ecology, which can lead consequently to habitat loss (Sidhoum et al. 2020). Bacterial communities and other microbiological indicators are important for biodegradation. They are frequently regarded as reliable bioindicators and ecological markers for environmental conditions due to their diversity and activity (Hwang et al. 2021).

Aquatic equilibrium is disrupted by anthropogenic factors that turn the acceptor medium into sewers, such as the direct release of wastewater into the environment. At such locations, pollution can cause the extinction of almost all living organisms (Aib et al. 2024). Water bodies, including wetlands, are subject to diverse physicochemical and microbiological conditions. Variables such as electrical conductivity, total nitrogen, total suspended solids, turbidity, and microbiological markers (Aib et al. 2024) provide significant insights into water quality and ecosystem health. According to recent research, wetlands exposed to household and industrial discharges exhibit high levels of heavy metal contamination, microbial community changes, and ecological degradation (Sidhoum et al. 2020). In specific region, estuaries have been turned into sewage dump sites due to heavy metal pollution, for example in Quanzhou Bay, which has also severely upset the ecological balance (Mei et al. 2024; Meli et al. 2014; Yang et al. 2020). Wetland ecosystem functioning and biodiversity are also seriously threatened by increased nitrogen inputs from agriculture and the burning of fossil fuels (Chandra et al. 2020). Microbial populations assist as the most sensitive and rapid bioindicators of environmental alterations, including microbiological changes (Urakawa and Bernhard 2017). Wetland biological assessment and bioindication concepts depend comprehensively on physicochemical and microbiological parameters (de Mello et al. 2025; Shi et al. 2024; Sims et al. 2013). Anthropogenic pressures are posing a growing threat to these ecosystems, especially in developing nations where wetland water quality has been seriously harmed by urbanization, agricultural runoff, and untreated wastewater discharge (MEA, 2005; Finlayson et al., 2018). Wetlands close to agricultural and industrial areas have been shown to exhibit nutrient enrichment, microbial contamination, and heavy metal accumulation in studies conducted worldwide (Ferreira et al. 2024; Giri and Qiu 2016). Many wetlands are not sufficiently protected, and there is still little long-term monitoring in place despite their ecological importance, especially in North African regions. In this context, a comparative regional assessment is crucial for evaluating pollution patterns and informing effective wetland management strategies.

Wetland ecosystems in Algeria are shaped by climatic, geographic, and human-induced factors. To capture these influences, we compared three regions: coastal, highland, and desert. The coastal wetlands are strongly influenced by marine dynamics and urbanization. The highland wetlands are shaped by altitude and agriculture. In contrast, the desert wetlands are affected by arid conditions and water scarcity (Finlayson and Spiers (1999); Ramsar Convention 2013). Despite the ecological importance of Algerian wetlands, comparative studies across multiple wetland systems remain scarce. This gap limits our understanding of regional environmental dynamics and hinders meaningful comparison with other studies conducted in Algeria.

The objective of our comparative study is to assess the physicochemical and microbiological properties of Algerian wetlands, with a particular emphasis on coastal regions, highland and the desert. By examining water quality parameters, we aim to identify pollution sources, assess seasonal variations, and highlight anthropogenic impacts. To achieve this, we employed principal component analysis (PCA) and correlation analysis to decipher the intricate connections between pollution drivers and wetland characteristics (Goshtasbi et al. 2022; Hwang et al. 2021). This study also aims to support efforts to preserve biodiversity, improve ecological resilience, and ensure the efficient management of wetland ecosystems in Algeria by establishing a comparative framework among coastal, highland, and desert wetlands. This framework will provide practical insights for ecosystem conservation and sustainable water resource management.

## Material and methods

### Site description

1. Coastal wetland (North)
  *Polluted wetland*: Boussedra Wetland (Annaba), it is a body of water that receives wastewater from the commune of El-Bouni (the largest commune in Algeria and still expanding) and from the El-Bouni industrial zone (flour mills, tile factories, mechanical engineering, food processing, etc.). These discharges are diverted without prior treatment (Boudraa et al., 2014, 2015).
  *Non-polluted wetland*: Garaet Hadj-Tahar Wetland (Skikda), this is a body of water used to irrigate the riparian farmers’ market garden crops (especially Cucurbitaceae; watermelons and yellow melons). It is unpolluted (Metallaoui and Houhamdi 2008, 2010).
2. Highland wetland (Mid)
  *Polluted wetland*: Sebkhet Bazer-Sakra (Sétif), is part of a high-altitude wetland eco-complex. It is a shallow, permanent saltwater body with salinity ranging from 0.22 to 0.43 mg/L MgCl₂. It is a highly polluted body of water. It collects industrial wastewater from Algeria's three electronic and electro-technical industrial poles (Sétif, El-Eulma and Bordj Bou Arreridj) (Baaziz et al., 2011).
  *Non-polluted wetland*: The Gadaïne eco-complex (Batna), it consists of five sub-chotts: Draâ Boultif, Teniet Saïda, Taricht, Saboune, and Gamra. These chotts are salt lakes typical of North Africa. The wetland is primarily fed by rainwater from Oued El Madher and Oued Zana. The water is brackish, with an alkaline pH. The region has an average temperature of 31 °C and annual precipitation of 334.5 mm. It is an non-polluted or very slightly polluted body of water. It receives rainwater and snowwater streams from the Aurès and Saharan Atlas Mountains (Marref et al., 2023).
3. Desert wetland (South)
  *Polluted wetland*: Chott Oum Raneb (Ouargla) is a permanent wetland in the northern Sahara, located near Sidi Kouiled and fed by wastewater from five settlements in the Ouargla region. It is a highly polluted Saharan water body. It receives untreated wastewater from the three major communes of Ouargla, Sidi Khouiled and Ain Beida, as well as irrigation water from palm groves (Houhamdi et al., 2025).
  *Non-polluted wetland*: Ayata Wetland (El-Méghair) is a body of water that receives irrigation water from the palm groves located upstream of the Oued Righ valley, which diversify into Chott Merouane (Megrerouche et al. 2025; Redaounia et al. 2025). Overflows from the Oued Righ canal have created this wetland and other smaller bodies of water. Water quality is good to slightly poor (Houhamdi et al., 2008, Nouidjem et al., 2012, Bensaci et al., 2013). Historically, it was the bed of an ancient Cretaceous Sea, later reclaimed by human activity in the late nineteenth century (Encyclopædia Britannica, 1878). The site plays a crucial role in maintaining Mediterranean and Central Saharan biodiversity (Biad et al., 2022). The upper basin harbors algae and phanerogams, while the lower basin contains sparse vegetation of grasses and halophilic algae.

**Fig. 1:**
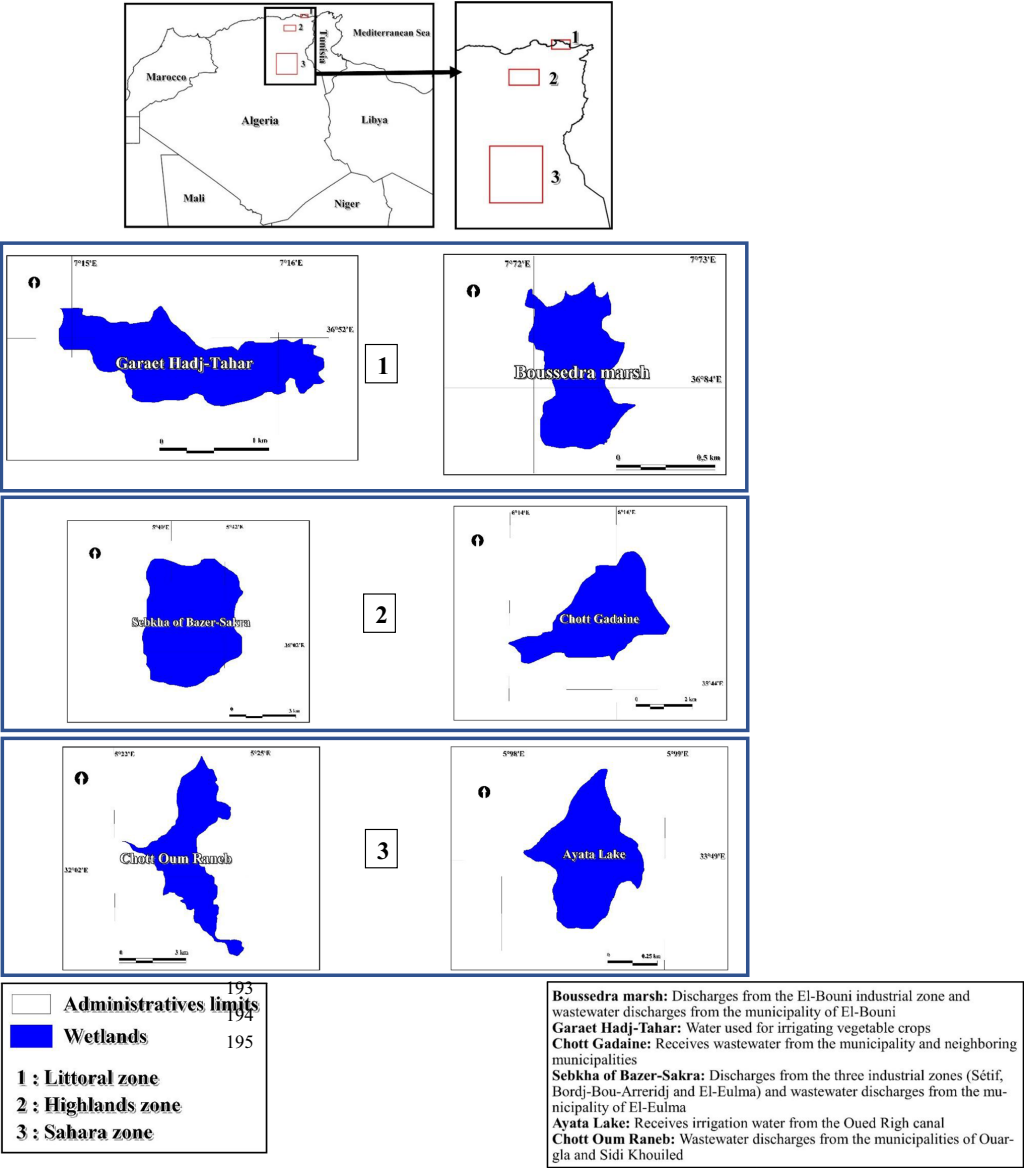
Geographical distribution of assessed wetlands in Algeria across different bioclimatic zones

Table 1 summarizes the geographic, climatic, and pollution-related characteristics of the six assessed wetland sites across Algeria. It highlights their coordinates, bioclimatic zones, Ramsar status, and pollution sources, offering context for understanding regional differences in water quality. Figure 1 illustrates the geographic distribution of the six studied wetlands across Algeria’s bioclimatic zones. This visual representation aids in contextualizing the environmental and anthropogenic pressures that may influence water quality in each region.

**Table 1:** Characteristics and pollution sources of assessed wetlands in Algeria

Site	Coordinates	Province	Bioclimatic zone	Area (ha)	Depth (m)	Ramsar status	Altitude (m)
Boussedra Wetland	36°50′40.88′′N, 7°43′37.30′′E	Annaba	Sub-humid	55	0.5–2.5	–	5
Garaet Hadj-Tahar	36°51′50′′N, 7°15′57′′E	Skikda	Sub-humid	102	0.5–2	2/1/2001	16
Chott Gadaines	35°44′ N, 6°14′ E	Batna	Semi-arid	350	0.2–1	–	813
Sebkhet Bazer-Sakra	36°02′56″ N, 5°41′02″ E	Sétif	Semi-arid	1550	0.3–0.8	12/12/2004	910–917
Ayata Wetland	30°30′20"N, 02°55′34′′E	El-Méghair	Arid (Saharan)	18,950	0.5–1.2	12/12/2004	367–478
Chott Oum Raneb	32°02′20″N, 5°23′31″E	Ouargla	Arid (Saharan)	1200	5–9	12/12/2004	126

### Sample collection and design

Sampling was carried out monthly between November 2021 and October 2022, yielding 12 replicates per station and capturing a full annual cycle. Six wetlands located across Algeria’s coastal (north), highland (central), and desert (south) regions were included in the study. Two permanent shoreline sampling stations (S1 and S2) were placed at comparable depths and at equal distances from the shoreline in each wetland to ensure spatial consistency. Although equidistant, the stations represented contrasting levels of anthropogenic influence: S1 was positioned near the principal pollution input (e.g., agricultural runoff, domestic discharge, or livestock activity), whereas S2 was oriented toward a less disturbed zone with minimal direct human impact. This design allowed water quality differences to be interpreted relative to a controlled pollution gradient rather than unequal spatial positioning. This systematic protocol resulted in 24 samples per station (48 per wetland) over the annual cycle.

A monthly grab sample was taken from each station in an effort to reduce temporal heterogeneity. These were divided into four traditional seasons: Fall (September–November), Winter (December–February), Spring (March–May), and Summer (June–August). This resulted in six total samples per wetland per season, with three temporal replicates per station. In order to avoid cross-contamination, sterile equipment was used for all sampling. Immediately after collection, the water was transferred to the lab for analysis in temperature-controlled, labeled containers. Both biological replicates (station-level samples) and technical replicates (lab duplicates for each parameter) were incorporated, following EPA wetland monitoring standards, to enhance data reliability and capture intra-site variability (EPA 2008, 2010, 2013).

### Physicochemical and microbiological analysis

A total of 25 parameters were evaluated, at the Laboratory of Biology, Water and Environment, University of 8 Mai 1945, Guelma, Algeria. Physicochemical, microbiological, and heavy metal parameters were analyzed to evaluate the water quality across the chosen study sites. The analyses were carried out following standardized methodologies (ISO and APHA methods) to guarantee accuracy and reproducibility. For physicochemical parameters, spectrophotometric techniques and calibrated instruments were used to measure pH, turbidity, electrical conductivity, dissolved oxygen, suspended solids, nutrients, and organic matter. Titration and ion chromatography were used to measure the major ions, including calcium, magnesium, potassium, chlorides, and sulfates, as well as the hardness of the water. Additionally, heavy metals such as nickel, manganese, copper, iron, and lead were also examined. Microbiological quality was assessed through membrane filtration and culture-based techniques, quantifying total germs, total coliforms, fecal coliforms, and fecal streptococci. Analyses were carried out in triplicate, and calibration curves were created and validated using certified reference materials to guarantee the reliability of the data. A thorough evaluation of the water quality at all the sites under study was made possible by these approaches.

### Climatic data collection

To account for the influence of climatic conditions on water quality, regional meteorological data were integrated into the analysis. Monthly values of air temperature (°C), precipitation (mm), and potential evaporation (mm) for each wetland region (coastal, highland, and desert) were obtained from the Algerian National Office of Meteorology (ONM) for the study period (November 2021–October 2022). These variables were selected due to their well-documented effects on hydrological balance, nutrient dynamics, and microbial activity in wetland ecosystems.

Temperature was considered important for driving biological metabolism and influencing dissolved oxygen levels, while rainfall affects dilution, runoff transport, and nutrient loading. Evaporation was incorporated to reflect concentration effects in arid conditions, particularly relevant to southern wetlands. Climatic variables were used to contextualize seasonal patterns observed in the physicochemical and microbiological datasets and to support the interpretation of PCA and correlation analyses.

### Statistical analysis

All statistical analyses were conducted using Python v3.11. To determine the suitability of statistical methods, the Shapiro–Wilk test was applied to assess the normality of the dataset, and histograms with normal distribution curves were generated for each region to visualize variance distribution. Since several parameters did not meet the assumptions of normality and homogeneity of variances, non-parametric tests were used for further comparisons.

We categorized the samples into six groups based on region (North, Mid, South) and pollution status (Polluted, Non-polluted). This resulted in:

- North (Polluted), North (Non-polluted)
- Mid (Polluted), Mid (Non-polluted)
- South (Polluted), South (Non-polluted)
- Mann–Whitney *U* test

This test was applied to compare each water quality parameter between polluted and non-polluted sites within the same region, for example:

- North: Garaet Hadj-Tahar vs. Boussedra Wetland
- Mid: Chott Gadaines vs. Sebkhet Bazer-Sakra
- South: Chott Oum Raneb vs. Ayata Wetland

This allowed us to assess the direct impact of pollution on water quality within each region.

2. Kruskal–Wallis Test + Dunn’s post hoc test

To examine differences across all six groups simultaneously (3 regions × 2 pollution levels), the Kruskal–Wallis test was used. This included pairwise comparisons like:

- North × Mid: Garaet Hadj-Tahar vs. Boussedra Wetland vs. Chott Gadaines vs. Sebkhet Bazer-Sakra
- North × South: Garaet Hadj-Tahar vs. Boussedra Wetland vs. Chott Oum Raneb vs. Ayata Wetland
- Mid × South: Chott Gadaines vs. Sebkhet Bazer-Sakra vs. Chott Oum Raneb vs. Ayata Wetland

Whenever the Kruskal–Wallis test showed a statistically significant result (*p* < 0.05), we conducted Dunn’s post hoc test to identify specific group differences. The goal was to detect whether:

- Polluted sites differ significantly from non-polluted sites, and
- Polluted sites in different regions show similar contamination profiles, as hypothesized.
- Seasonal comparison

To evaluate seasonal variability, the dataset was grouped into wet (November–April) and dry (May–October) periods, reflecting the regional rainfall pattern. Key nutrient parameters (nitrate, ammonium, phosphate) and microbial indicators (total germs, total coliforms, fecal coliforms) were compared between seasons. Non-parametric tests were applied where data did not follow a normal distribution, and seasonal distributions were visualized using boxplots to highlight differences between wet and dry periods.

4. Tests of suitability for principal component analysis (PCA) and results

To reduce dimensionality and identify the most influential parameters for regional comparison, we conducted a PCA. Prior to PCA, all variables were standardized to a scale between − 1 and 1. From the full set of 25 measured parameters, a refined subset of 15 key physicochemical and microbiological variables was selected for PCA to reduce dimensionality and highlight the dominant gradients structuring water quality variation. PCA was applied after verifying data suitability through the Kaiser–Meyer–Olkin (KMO) measure and Bartlett’s test of sphericity. The overall KMO value was 0.84, indicating meritorious sampling adequacy, while regional KMOs were 0.55 for the North (mediocre), 0.76 for the Mid (middling), and 0.86 for the South (meritorious). Bartlett’s test was highly significant in all cases (All: χ^2^ = 3806, *p* < 0.001; North: χ^2^ = 1370, *p* < 0.001; Mid: χ^2^ = 1874, *p* < 0.001; South: χ^2^ = 1653, *p* < 0.001), confirming that correlations were sufficient for factor extraction. Two components were retained based on eigenvalues greater than 1 and inspection of the scree plot. Communalities showed that most variables were adequately represented by the factor solution, with key physicochemical and microbiological parameters (e.g., pH, electrical conductivity, organic matter, coliform indicators, and major ions) exhibiting communalities above the 0.30 retention threshold. Trace metals, particularly copper, iron, lead, and manganese, displayed very high communalities (> 0.75), indicating strong contributions to the second component. Although a few variables such as magnesium and turbidity had lower communalities (< 0.25), they were retained due to their ecological and regulatory relevance. Overall, the first component primarily reflected organic and ionic pollution gradients, while the second component captured the variability associated with dissolved trace metals.

5. Spearman correlation

Finally, Spearman correlation analysis was used to evaluate the relationships between different parameters in all wetland sites collectively and each region separately.

A significance level of *p* < 0.05 was set for all statistical tests, and results are reported as test statistics with corresponding *p* values.

## Results

### Data distribution and descriptive analysis

The Shapiro–Wilk test and histogram curve fitting (Fig. 1) showed that most parameters were not normally distributed (*p* < 0.05), requiring non-parametric tests. Descriptive statistics (medians and ranges) were generated for polluted and non-polluted sites across coastal, highland, and desert wetlands. Polluted sites consistently had higher turbidity, electrical conductivity, nutrients (e.g., ammonium, nitrates), and heavy metals (e.g., lead, copper, iron), reflecting strong anthropogenic influence. In contrast, non-polluted sites exhibited lower values and less variability, indicating better water quality. Among coastal wetlands, Boussedra showed elevated pollution levels, while Garaet Hadj-Tahar appeared more stable, with fluctuations mainly linked to natural processes. These findings emphasize the role of turbidity, conductivity, trace metals, and microbial counts as key indicators of wetland water quality (Table 1).

**Fig. 2:**
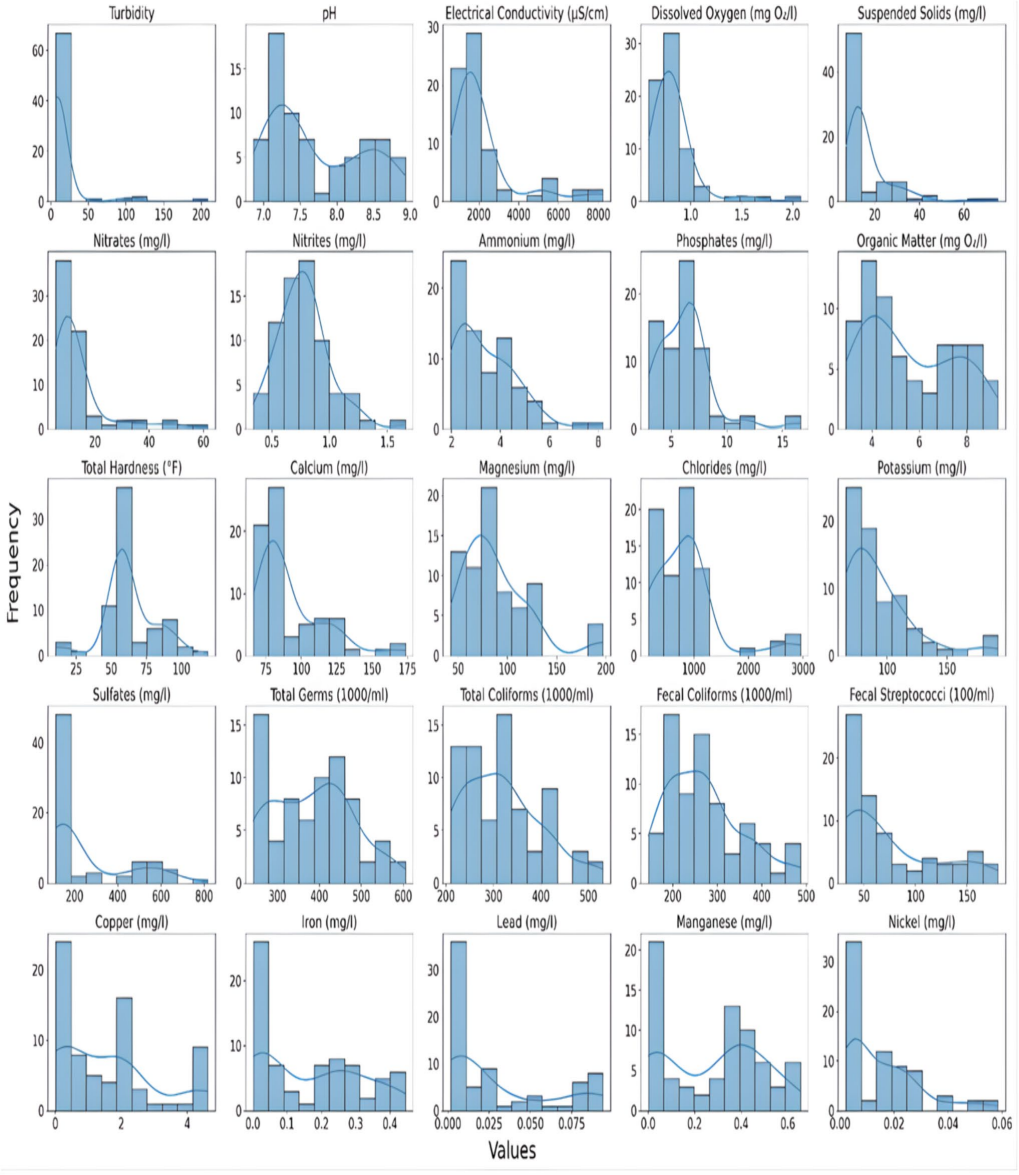
Histograms of water quality parameters across all the different wetlands assessed

Table 2 presents the descriptive statistics of physicochemical, microbiological, and heavy metal parameters for the northern (coastal) region. This comparison between the polluted Boussedra Wetland and non-polluted Garaet Hadj-Tahar highlights differences in water quality, suggesting significant anthropogenic influence at the former site (Fig. 2).

**Table 2:** Descriptive statistics of water quality parameters in the northern region

	Boussedra Wetland			Garaet Hadj-Tahar				
	Mean	Min	Max	SD	Mean	Min	Max	SD
Turbidity	9.29	7.89	10.9	1.02	7.68	6.79	8.86	0.82
pH	7.3	7.02	7.6	0.18	7.66	6.86	8.75	0.73
Electrical,C(µS/cm)	1517.82	592.9	1848.33	383.12	1310.77	1235.69	1404	63.82
D,Oxygen (mg O₂/l)	0.73	0.6	0.86	0.09	0.76	0.58	0.93	0.1
Suspended,S (mg/L)	12.23	11.22	12.82	0.49	12.85	11.66	13.8	0.68
Nitrates (mg/L)	8.44	7.75	9.44	0.48	13.25	12.6	13.7	0.4
Nitrites (mg/L)	0.8	0.55	1.18	0.18	0.76	0.49	0.95	0.15
Ammonium (mg/L)	2.41	1.96	3.55	0.46	4.52	2.35	5.54	0.95
Phosphates (mg/L)	4.35	2.94	6.58	1.16	6.72	6	7.59	0.5
O. Matter (mgO₂/l)	3.77	2.9	4.75	0.62	5.8	3.25	7.34	1.16
T Hardness (°F)	57.36	50.1	62.75	3.87	54.63	51.44	58.06	2.57
Calcium (mg/L)	77.67	69.82	87.14	4.96	85.01	82.12	88.06	1.96
Magnesium (mg/L)	83.66	42.55	124.18	25.26	57.1	54	61.2	2.27
Chlorides (mg/L)	794.42	168.5	1207	384.15	941.41	505.94	1019.5	141.71
Potassium (mg/L)	72.83	66	88	7.66	93.46	81.5	107	8.57
Sulfates (mg/L)	140.96	112.5	181.5	20.36	141.25	122.5	165.5	13.87
T. Germs (1000/ml)	315	242.5	407.5	57.39	457.54	366.5	554	58.64
Total, C (1000/ml)	270	210	335	48.16	330.96	287	412	38.15
Fecal. C (1000/ml)	231.38	169	295	49.82	260.25	197.5	304.5	27.4
Fecal, S (100/ml)	49.79	32.5	73	16.97	75.88	32.5	158	48.04
Copper (mg/L)	2	1.64	2.48	0.22	0.92	0.79	1.1	0.08
Iron (mg/L)	0.22	0.18	0.3	0.03	0.05	0.01	0.09	0.03
Lead (mg/L)	0.02	0.02	0.02	0	0.01	0	0.01	0
Manganese (mg/L)	0.4	0.31	0.51	0.06	0.24	0.13	0.4	0.09
Nickel (mg/L)	**0.02**	**0.01**	**0.02**	**0**	**0.01**	**0**	**0.01**	**0**

Table 2 summarizes key metrics such as electrical conductivity, turbidity, pH, dissolved oxygen, suspended solids, and microbial indicators. Significant differences were observed between the two highland wetlands. The polluted Sebkhet Bazer-Sakra showed elevated turbidity, conductivity, nutrients (nitrates, ammonium, phosphates), and microbial contamination (total germs, coliforms, fecal coliforms) compared to the uncontaminated Gadaïne Wetland, which exhibited consistently lower values. Table 3 further highlights these contrasts, with higher turbidity, nutrient loads, and fecal indicators in Sebkhet Bazer-Sakra, reflecting strong anthropogenic pressures from agriculture and urban effluents. These results confirm the degraded water quality of the polluted site and underline the vulnerability of highland wetlands to human activities.

**Table 3:** Descriptive statistics of water quality parameters in the middle region

	Sebkhet Bazer-Sakra			Wetland Gadaines				
	Mean	Min	Max	SD	Mean	Min	Max	SD
Turbidity	54.24	6.69	208.83	63.52	1.51	1.93	10	7.89
pH	8.4	8	8.88	0.26	0.16	− 0.52	7.21	7.02
Electrical,C(µS/cm)	4378.31	1616.88	8228.47	2699.87	0.4	− 1.8	1342.66	1084.2
D,Oxygen (mg O₂/l)	1.23	0.88	2.08	0.37	1.27	0.94	0.74	0.6
Suspended,S (mg/L)	22.25	7.07	75.16	18.11	2.56	7.64	12	11.22
Nitrates (mg/L)	32.65	9.43	61.66	16.56	0.13	− 0.82	14.16	11.5
Nitrites (mg/L)	0.95	0.56	1.66	0.32	1.01	0.69	0.83	0.55
Ammonium (mg/L)	4.47	2.38	8.19	1.87	0.78	− 0.2	2.82	2.1
Phosphates (mg/L)	8.55	3.88	16.61	4.41	0.93	− 0.51	6.12	4.15
O. Matter (mgO₂/l)	7.25	5.73	8.45	0.8	− 0.29	− 0.13	4.5	3.65
T Hardness (°F)	86.5	69	119	14.98	0.92	0.41	59.21	50.1
Calcium (mg/L)	130.65	100.24	173.55	22.6	0.8	− 0.26	74.78	66.1
Magnesium (mg/L)	114.67	58.82	196.94	61.28	0.55	− 1.83	107.25	52.28
Chlorides (mg/L)	1341.22	260.66	2951.18	1089.8	0.46	− 1.86	637.92	168.5
Potassium (mg/L)	127.92	65.5	192.5	45.14	− 0.01	− 1.38	79.46	66
Sulfates (mg/L)	444.41	246.5	817.5	184.79	0.67	− 0.42	146.17	112.5
T. Germs (1000/ml)	420.29	268	605	136.72	0.1	− 1.99	353.62	242.5
Total, C (1000/ml)	369.83	242.5	530.5	128.66	0.14	− 2.23	274.79	210
Fecal. C (1000/ml)	334.54	197.5	487	126.88	0.13	− 2.21	209.54	145
Fecal, S (100/ml)	79.33	34	126	30.14	0.22	− 1.32	41.83	32.5
Copper (mg/L)	4.2	3.18	4.62	0.49	− 1.28	0.52	0.08	0.03
Iron (mg/L)	0.4	0.34	0.44	0.03	− 0.11	0.24	0.01	0
Lead (mg/L)	0.09	0.08	0.09	0	0.22	− 1.51	0	0
Manganese (mg/L)	0.57	0.46	0.66	0.06	− 0.36	− 0.35	0.05	0
Nickel (mg/L)	**0.03**	**0.02**	**0.04**	**0.01**	**1.48**	**3.16**	**0**	**0**

Chott Oum Raneb shows severe pollution, with high chemical and microbial contamination, while Lac El-Goléa maintains relatively better water quality despite elevated potassium and sulfates (Tables 3 and 4). Key differences appear in suspended solids, organic matter, phosphates, and microbial indicators. Heavy metals and microbial loads in Chott Oum Raneb point to untreated sewage and industrial discharge, highlighting the fragile desert wetland ecosystem and the urgent need for pollution control.

**Table 4:** Descriptive statistics of water quality parameters in the desert region

	Chott Oum Raneb			Lac El-Goléa				
	Mean	Min	Max	SD	Mean	Min	Max	SD
Turbidity	9.29	7.89	10.9	1.02	8.1	7.5	8.64	0.41
pH	7.3	7.02	7.6	0.18	8.47	7.86	8.93	0.34
Electrical,C(µS/cm)	1517.82	592.9	1848.33	383.12	3206.12	2192.69	5330.14	1258.89
D,Oxygen (mg O₂/l)	0.73	0.6	0.86	0.09	0.87	0.74	1	0.07
Suspended,S (mg/L)	12.23	11.22	12.82	0.49	33.54	16.49	62.13	11.63
Nitrates (mg/L)	8.44	7.75	9.44	0.48	7.34	5.5	8.35	0.84
Nitrites (mg/L)	0.8	0.55	1.18	0.18	0.52	0.34	0.69	0.13
Ammonium (mg/L)	2.41	1.96	3.55	0.46	4.01	3.7	4.47	0.28
Phosphates (mg/L)	4.35	2.94	6.58	1.16	7.81	6.75	11.04	1.23
O. Matter (mgO₂/l)	3.77	2.9	4.75	0.62	8.44	7.9	9.32	0.49
T Hardness (°F)	57.36	50.1	62.75	3.87	63.59	10.33	96.5	36.18
Calcium (mg/L)	77.67	69.82	87.14	4.96	106.72	86.2	126.34	14.02
Magnesium (mg/L)	83.66	42.55	124.18	25.26	84.02	75.44	97.2	6.35
Chlorides (mg/L)	794.42	168.5	1207	384.15	686.08	333.5	2932.52	712.98
Potassium (mg/L)	72.83	66	88	7.66	112.42	97.14	139.55	12.7
Sulfates (mg/L)	140.96	112.5	181.5	20.36	554.5	470	669.11	67.69
T. Germs (1000/ml)	315	242.5	407.5	57.39	443.75	381	476	27.61
Total, C (1000/ml)	270	210	335	48.16	401.71	355	430	25.38
Fecal. C (1000/ml)	231.38	169	295	49.82	365	320	399.5	25.05
Fecal, S (100/ml)	49.79	32.5	73	16.97	152.96	112	180	19.72
Copper (mg/L)	2.02	1.37	2.33	0.32	0.09	0.02	0.2	0.06
Iron (mg/L)	0.28	0.24	0.32	0.02	0.05	0.01	0.13	0.05
Lead (mg/L)	0.05	0.03	0.08	0.02	0	0	0.01	0
Manganese (mg/L)	0.41	0.34	0.47	0.04	0	0	0.01	0
Nickel (mg/L)	**0.04**	**0.02**	**0.06**	**0.01**	**0**	**0**	**0.01**	**0**

### Differences between polluted and non-polluted sites

The Mann–Whitney *U* test reveals significant differences in water quality parameters across the North, Mid, and South regions, reflecting environmental influences such as geographic variation and industrial impact. In the North, turbidity, electrical conductivity, and suspended solids vary notably, while in the South, almost all parameters are significant. Non-significant parameters, such as pH and dissolved oxygen in the North or magnesium and chlorides in the South, suggest environmental or anthropogenic similarities. These patterns highlight how factors like climate, industrial activity, geology, and regulation shape regional water quality.

**Fig. 3:**
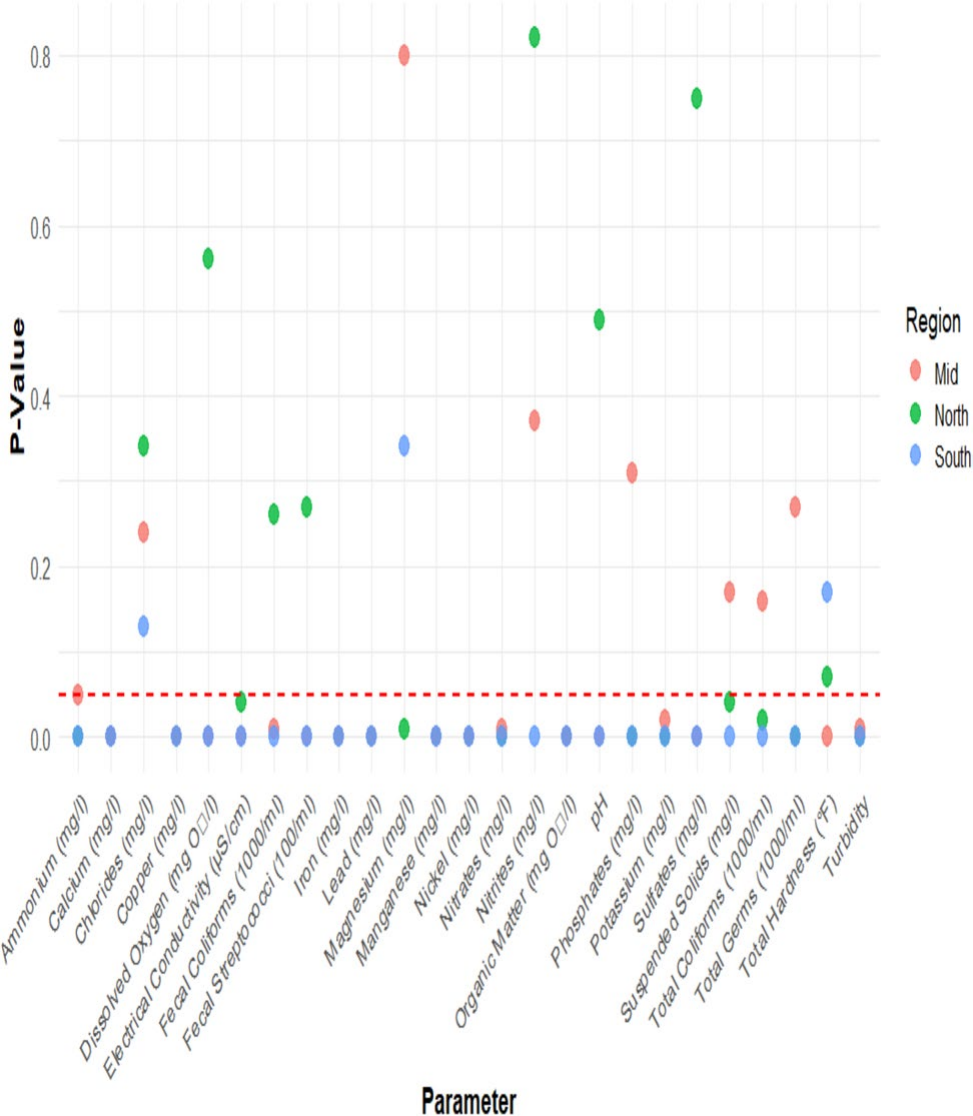
Results of Mann–Whitney test of water quality parameters across regions

The analysis (Fig. 3) revealed significant differences between polluted and non-polluted sites in physicochemical, nutrient, heavy metal, and microbiological parameters. Parameters with *p* < 0.05 indicate statistically significant differences, highlighting the impact of pollution versus natural factors. Polluted sites showed higher turbidity, electrical conductivity, organic matter, nutrients (nitrates, ammonium, phosphates), and heavy metals (lead, copper, iron), while dissolved oxygen was lower, reflecting organic pollution, agricultural runoff, and industrial discharge. Magnesium, chlorides, and nitrites showed no significant differences, suggesting geochemical influences or localized variability. Fecal streptococci were elevated in contaminated sites, indicating untreated sewage input, whereas total and fecal coliforms were not significant, likely due to hydrological variability and environmental conditions. These results underscore the combined influence of natural and anthropogenic factors on water quality.

Numerous exceedances of Algerian and WHO environmental standards were found when the observed water quality parameters were compared across the contaminated wetlands. Nitrate concentrations in the mid-region (up to 66 mg/L) and phosphate levels across all sites (up to 15.7 mg/L) exceeded the recognized limits by a considerable amount of 50 mg/L and 0.1 mg/L, respectively. Ammonium levels also consistently exceeded the 0.5 mg/L threshold, especially in the southern and mid-regions. Moreover, heavy metals such as lead (0.097 mg/L), iron (0.42 mg/L), manganese (0.82 mg/L), and nickel (0.056 mg/L) were detected in levels significantly higher than allowed limits, which could be harmful to the environment and public health. There was a significant amount of microbiological contamination, with levels of fecal streptococci and coliforms surpassing acceptable thresholds for recreation and irrigation. Electrical conductivity values reached as high as 9250 µS/cm in the mid-region, indicating elevated salinity (Table 5).

**Table 5:** Comparison of observed values with WHO and Algerian Water Quality Standards

Parameters	Observed Range (mg/L or CFU/100 ml)	WHO limit	Algerian Standard	Exceeds Standard?
Nitrate (NO₃⁻)	3.78–66	50 mg/L	30 mg/L	Yes (up to 66)
Ammonium (NH₄⁺)	1.03–8.5	0.5 mg/L	1 mg/L	Yes (up to 8.5)
Phosphate (PO₄^3^⁻)	1.3–15.7	< 0.1 mg/L	0.1 mg/L	Yes (up to 15.7)
Fecal coliforms	120–430 (1000/ml → CFU/100 ml)	< 100 CFU/100 ml	1000 CFU/100 ml	Yes (irrigation unsafe)
Streptococci (fecal)	18–75 CFU/100 ml	< 20 CFU/100 ml^a^	Not specified	Yes (up to 75)
Electrical Conductivity	1024–9250 µS/cm	1400 µS/cm	3000 µS/cm	Yes (especially desert)
Lead (Pb)	0.011–0.097 mg/L	0.01 mg/L	0.01 mg/L	Yes (all sites)
Iron (Fe)	0.16–0.42 mg/L	0.3 mg/L	0.3 mg/L	Yes (many sites)
Manganese (Mn)	0.3–0.82 mg/L	0.1 mg/L	0.1 mg/L	Yes
Copper (Cu)	1.33–4.65 mg/L	2.0 mg/L	2.0 mg/L	Yes (mid region mainly)
Nickel (Ni)	0.01–0.056 mg/L	0.02 mg/L	0.02 mg/L	Yes

^a^WHO does not have official fecal streptococci limits, but recreational guidelines suggest < 20 CFU/100 ml for low health risk (JORADP 2012; WHO 2008)

Polluted wetlands exhibit widespread exceedances when measured water quality parameters are compared to Algerian standards (Table 6). Turbidity, dissolved oxygen, ammonium, phosphates, and microbial indicators all routinely exceeded thresholds at Boussedra, Sebkhet Bazer-Sakra, and Chott Oum Raneb, confirming their designation as polluted sites. The non-polluted wetlands Garaet Hadj-Tahar, Gadaïne, and Ayata mostly met the standard, though occasional exceedances were still noted, especially for phosphate concentrations and microbial counts.

**Table 6:** Comparison of mean water quality values from Algerian wetlands with Algerian standards

Parameter	Algerian standard	Boussedra wetland	Garaet Hadj Tahar	Sebkhet Bazer-Sakra	Chott Gadaines	Chott Oum Raneb	Ayata Wetland	Compliance notes
Turbidity (NTU)	< 5	9.29 ✗	7.68 ✗	54.24 ✗	1.51 ✓	9.29 ✗	8.1 ✗	Exceeded in all but Gadaïne
pH	6.5–8.5	7.3 ✓	7.66 ✓	8.4 ✓	7.0 ✓	7.3 ✓	8.47 ✓	All within
DO (mg/L)	> 5	0.73 ✗	0.76 ✗	1.23 ✗	1.27 ✗	0.73 ✗	0.87 ✗	All far below standard
Nitrate (mg/L)	< 50	8.44 ✓	13.25 ✓	32.65 ✗ (EPA)	0.13 ✓	8.44 ✓	7.34 ✓	Sebkhet exceeds EPA
Ammonium (mg/L)	< 0.5	2.41 ✗	4.52 ✗	4.47 ✗	1.01 ✗	2.41 ✗	4.01 ✗	Exceeded in all
Phosphate (mg/L)	< 0.5	4.35 ✗	6.72 ✗	8.55 ✗	0.78 ✗	4.35 ✗	7.81 ✗	Exceeded in all
Total coliforms (CFU/100 ml)	0	270,000 ✗	330,960 ✗	369,830 ✗	140 ✓	270,000 ✗	401,710 ✗	All exceed except Gadaïne
Fecal Coliforms (CFU/100 ml)	0	231,380 ✗	260,250 ✗	334,540 ✗	130 ✓	231,380 ✗	365,000 ✗	All exceed except Gadaïne
Lead (mg/L)	< 0.01	0.02 ✗	0.01 ✓	0.09 ✗	0.22 ✗	0.05 ✗	0 ✓	Exceeds in most
Copper (mg/L)	< 1–2	2 ✓	0.92 ✓	4.2 ✗	−1.28 (?)	2.02 ✓	0.09 ✓	Sebkhet exceeds
Nickel (mg/L)	< 0.02	0.02 ✓	0.01 ✓	0.03 ✓	1.48 ✗	0.04 ✓	0 ✓	Gadaïne anomaly (1.48 mg/L)

### Regional differences in water quality

The findings in Table 7 compares the results of the Kruskal–Wallis test between three distinct regions. For clarity, the variables are arranged according to their type. Reports of significant regional differences are based on p-values (significance at *p* < 0.05).

**Table 7:** Non-parametric statistical evaluation of water quality across different pollution levels

Parameter	Pollution level	Test statistic	*P* value
Physicochemical parameters			
Turbidity (NTU)	Polluted	10.39	0.01*
	Non-polluted	16.09	0*
pH	Polluted	23.44	0*
	Non-polluted	16.43	0*
Electrical conductivity (µS/cm)	Polluted	15.18	0*
	Non-polluted	23.65	0*
Dissolved oxygen (mg O₂/L)	Polluted	23.41	0*
	Non-polluted	11.4	0*
Suspended Solids (mg/L)	Polluted	2.6	0.27
	Non-polluted	27.42	0*
Nitrates (mg/L)	Polluted	22.74	0*
	Non-polluted	23.63	0*
Nitrites (mg/L)	Polluted	1.9	0.39
	Non-polluted	17.45	0*
Ammonium (mg/L)	Polluted	15.18	0*
	Non-polluted	21.43	0*
Phosphates (mg/L)	Polluted	13.16	0*
	Non-polluted	10.25	0.01*
Organic matter (mg O₂/l)	Polluted	23.46	0*
	Non-polluted	27.11	0*
Total hardness (°F)	Polluted	23.39	0*
	Non-polluted	6.05	0.05*
Calcium (mg/L)	Polluted	23.41	0*
	Non-polluted	25.6	0*
Magnesium (mg/L)	Polluted	0.76	0.68
	Non-polluted	23.23	0*
Chlorides (mg/L)	Polluted	0.37	0.83
	Non-polluted	10.25	0.01*
Potassium (mg/L)	Polluted	11.75	0*
	Non-polluted	27.2	0*
Sulfates (mg/L)	Polluted	23.39	0*
	Non-polluted	23.56	0*
Microbiological parameters			
Total germs (1000/ml)	Polluted	4.48	0.11
	Non-polluted	9.67	0.01*
Total coliforms (1000/ml)	Polluted	4.07	0.13
	Non-polluted	23.29	0*
Fecal coliforms (1000/ml)	Polluted	5.54	0.06
	Non-polluted	26.47	0*
Fecal Streptococci (100/ml)	Polluted	6.7	0.04*
	Non-polluted	19.83	0*
Heavy metals			
Copper (mg/L)	Polluted	24.11	0*
	Non-polluted	23.36	0*
Iron (mg/L)	Polluted	28.48	0*
	Non-polluted	17.18	0*
Lead (mg/L)	Polluted	31.16	0*
	Non-polluted	4.74	0.09
Manganese (mg/L)	Polluted	21.86	0*
	Non-polluted	29.28	0*
Nickel (mg/L)	Polluted	25.16	0*
	Non-polluted	2.05	0.36

* indicates statistically significant difference (*p* < 0.05)

The Kruskal–Wallis test revealed significant regional differences (*p* < 0.05) in key water quality parameters across the coastal, highland, and desert wetlands. Significant variations were observed in turbidity, pH, electrical conductivity, dissolved oxygen, copper, and lead in the coastal region. In contrast to polluted sites, which showed increased levels of heavy metals like copper and lead, which were indicative of industrial and urban impacts, non-polluted sites had higher dissolved oxygen and lower turbidity, indicating better water quality.

In the highland region, nitrates, ammonium, organic matter, phosphates, and potassium showed significant differences (*p* < 0.05). According to these results, farming practices have a major impact on the deterioration of water quality in contaminated areas. At non-polluted locations, there was some indication of localized nutrient enrichment and moderate water quality. The desert region exhibited significant differences (*p* < 0.05) in electrical conductivity, turbidity, and manganese. Extreme turbidity and electrical conductivity values were exhibited by polluted sites, most likely because of restricted water flow and pollutant buildup. Heavy metal levels, especially manganese, varied significantly between sites as well.

### Seasonal variation in nutrients and microbial indicators

**Fig. 4:**
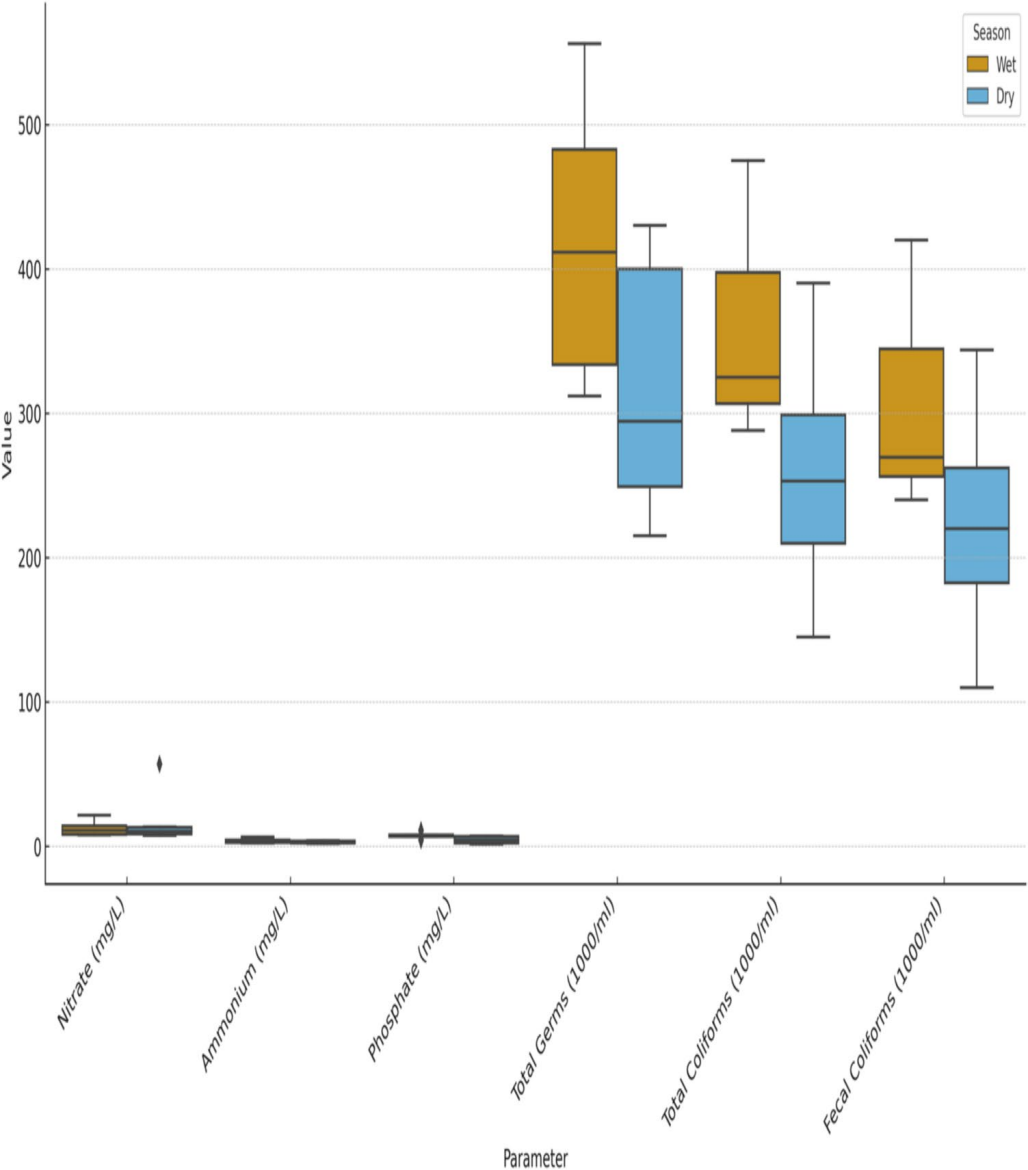
Seasonal variation in nutrient concentrations and microbial indicators across six Algerian wetlands, comparing wet and dry periods

Figure 4 compares the wet and dry seasons to show the seasonal variation in microbial indicators (total germs, total coliforms, and fecal coliforms) and nutrient concentrations (nitrate, ammonium, and phosphate) across the six wetlands under study. Overall, during the dry season, nutrient levels (especially those of phosphate and ammonium) tended to rise, most likely as a result of higher evaporation rates that concentrate pollutants and decreased dilution capacity. Microbial indicators, on the other hand, typically peaked during the rainy season, indicating increased runoff and pollutant inflows from urban and agricultural sources. This disparate pattern demonstrates the twofold impact of hydrological conditions: during the dry months, chemical loads are increased by water scarcity, while during the wet season, microbial contamination is increased by rainfall-driven inputs. These results demonstrate that wetland water quality is significantly influenced by seasonality, which is why monitoring and management initiatives need to take this into account.

### Comparative analysis of water quality parameters across wetlands in all regions

In contrast to our earlier strategy, which divided groups based on region, we combined polluted and non-polluted sites (across all regions) to perform Dunn’s post-hoc analysis. Chloride, which had a value of 9.77 and a *p* value of 0.08 (*p* > 0.05), was not included in the subsequent Dunn’s post hoc test because it lacked statistical significance. We used 24 of the 25 parameters that were available. The results are organized into three main groups: chemical parameters, microbiological parameters, and heavy metals. For each parameter, we describe the observed statistically significant differences in detail and talk about how they affect the environment.

1. Chemical parameters

Ammonium concentrations differed significantly between polluted and non-polluted sites across all regions, with notable differences in the North and South (*p* < 0.05). Even among non-polluted sites, variations between the Mid and North regions (*p* = 0.03) suggest natural influences such as vegetation, hydrology, or soil composition. Elevated ammonium levels likely result from wastewater discharge and agricultural runoff. Calcium was significantly higher in polluted sites of the Mid and South regions (*p* < 0.05), with marked differences between Mid (Polluted) and North (Non-polluted) (*p* = 0.03) and South (Non-polluted) (*p* = 0). Industrial effluents, agricultural lime, and local geology (e.g., limestone deposits) may explain these patterns, highlighting the need to consider both pollution and natural variability. Dissolved oxygen (DO) was significantly lower in polluted Mid and South sites (*p* < 0.05), particularly between South Non-polluted and Polluted (*p* = 0.05) and Mid Non-polluted and Polluted (*p* = 0). Reduced DO likely reflects higher organic loads promoting microbial oxygen consumption, while temperature, flow, and vegetation also influence regional variations. Electrical conductivity (EC) was higher in polluted Mid and South sites (*p* < 0.05), with significant differences between North (Polluted) and South (Non-polluted) and Mid (Non-polluted) and Mid (Polluted) (*p* = 0.01). Increased EC indicates higher dissolved ions from pollution or agricultural inputs, with local hydrology (e.g., groundwater inflow or evaporation) further affecting values, emphasizing the need for targeted monitoring. Polluted sites exhibited higher turbidity in the North and South (*p* = 0.1 and 0.34), likely due to runoff rich in organic matter and suspended solids. Hardness was elevated in polluted sites of the Mid and North regions (*p* = 0.06 and 0), likely from dissolved minerals of human origin, with regional differences also observed between North and South non-polluted sites (*p* = 0.04). Polluted Mid and South sites showed significantly higher levels of salts (*p* = 0.01 and 0), potassium (Mid and North, *p* = 0.05 and 0.03), and phosphates (Mid and North, *p* = 0.01 and 0.02), reflecting industrial and agricultural inputs and raising eutrophication concerns. Polluted Mid and South sites had lower pH (*p* = 0), while organic matter, nitrites, and nitrates were higher across regions, indicating sewage and fertilizer impacts. Magnesium was elevated in polluted North sites (*p* = 0.12), suggesting mineral contributions. These results show that both natural variability and human activities shape wetland water chemistry, emphasizing the need for region-specific water management strategies.

2. Microbiological parameters

Polluted sites in the South had significantly higher fecal streptococci than non-polluted sites (*p* = 0), with notable differences also between North and South non-polluted sites (*p* = 0.01), indicating human or animal waste contamination. Regional differences suggest that local land use and hydrology influence baseline microbial levels. Fecal coliforms were elevated in polluted Mid and South sites, with significant differences between South and Mid sites (*p* = 0.03), reflecting sewage or agricultural runoff. These findings highlight the need for region-specific strategies to manage microbial pollution effectively. Total germ counts were significantly higher in polluted sites, with notable differences between the North and South regions (*p* = 0), likely due to elevated organic and nutrient loads. Total coliforms were also higher in polluted South sites (*p* = 0) and differed between Mid and South non-polluted sites (*p* = 0), reflecting impacts from sewage, agricultural runoff, and animal waste. Overall, fecal streptococci, fecal coliforms, total germs, and total coliforms were elevated in polluted wetlands, demonstrating that pollution markedly increases microbial contamination. Regional variability suggests that both local environmental conditions and pollution sources shape microbial water quality. These findings underscore the public health importance of strict sanitation and waste management in impacted wetlands.

3. Heavy metals

North non-polluted sites had lower iron concentrations than North polluted sites (*p* = 0.28), while Mid and South polluted sites showed significantly higher iron levels (*p* = 0 and 0.01), likely from industrial discharges, mining, or natural weathering. These variations reflect both regional geology and pollution sources. Manganese was also higher in polluted Mid and South sites, with highly significant differences between non-polluted and polluted sites (*p* = 0), likely due to mineral weathering and industrial inputs. Nickel concentrations were elevated in polluted Mid (*p* = 0) and North (*p* = 0.04) sites, emphasizing the need for region-specific heavy metal management strategies. Pollution significantly elevates iron, manganese, and nickel levels in wetlands, driven by industrial discharges, mining, and human activities. These heavy metals, together with high nutrients and microbial contaminants, degrade water quality, threatening aquatic life and public health. Regional differences highlight the interplay of natural conditions and anthropogenic inputs. Effective mitigation requires region-specific strategies, including controlling industrial and agricultural runoff and promoting sustainable land use, alongside continuous monitoring of key indicators like turbidity to safeguard ecosystem health.

### Comparative correlation analysis of water quality parameters across wetland conditions

One effective method for comprehending the connections between various water quality parameters is to use correlation matrices. In this study, three distinct correlation analyses were carried out, one for the region’s wetlands, one for non-polluted wetlands, and one for the entire dataset (all regions). These analyses show how important parameters change in different environments and aid in the identification of possible monitoring proxy variables.

#### Polluted wetland analysis

**Fig. 5:**
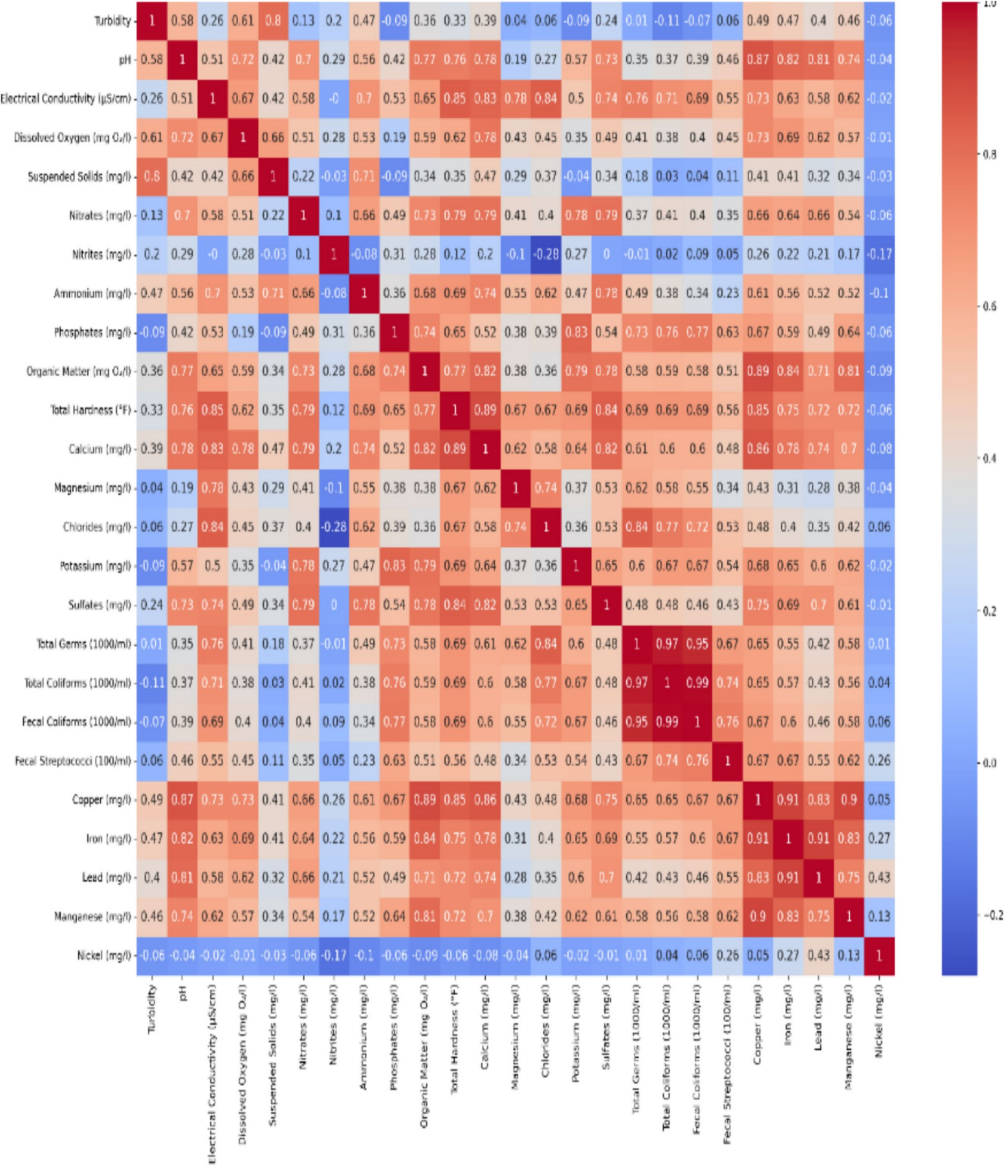
Polluted wetlands: correlation analysis of physicochemical and microbiological parameters

The correlation matrix reveals key relationships among water quality parameters. Diagonal values are 1.0, reflecting each parameter’s perfect correlation with itself, while off-diagonal values show significant pairwise correlations. Turbidity and pH are moderately negatively correlated (r = − 0.58), indicating pH decreases as turbidity rises. Electrical conductivity correlates positively with pH (r = 0.51) and strongly with calcium (r = 0.83), while total hardness also correlates strongly with calcium (r = 0.89), highlighting calcium’s role in conductivity and hardness. pH and organic matter show a strong positive correlation (r = 0.77), possibly due to alkaline release during decomposition. Total germs and total coliforms are extremely correlated (r = 0.97), supporting coliforms as reliable microbial indicators, and turbidity strongly correlates with suspended solids (r = 0.80), confirming suspended solids as a major turbidity driver (Fig. 5).

#### Non-polluted wetland analysis

**Fig. 6:**
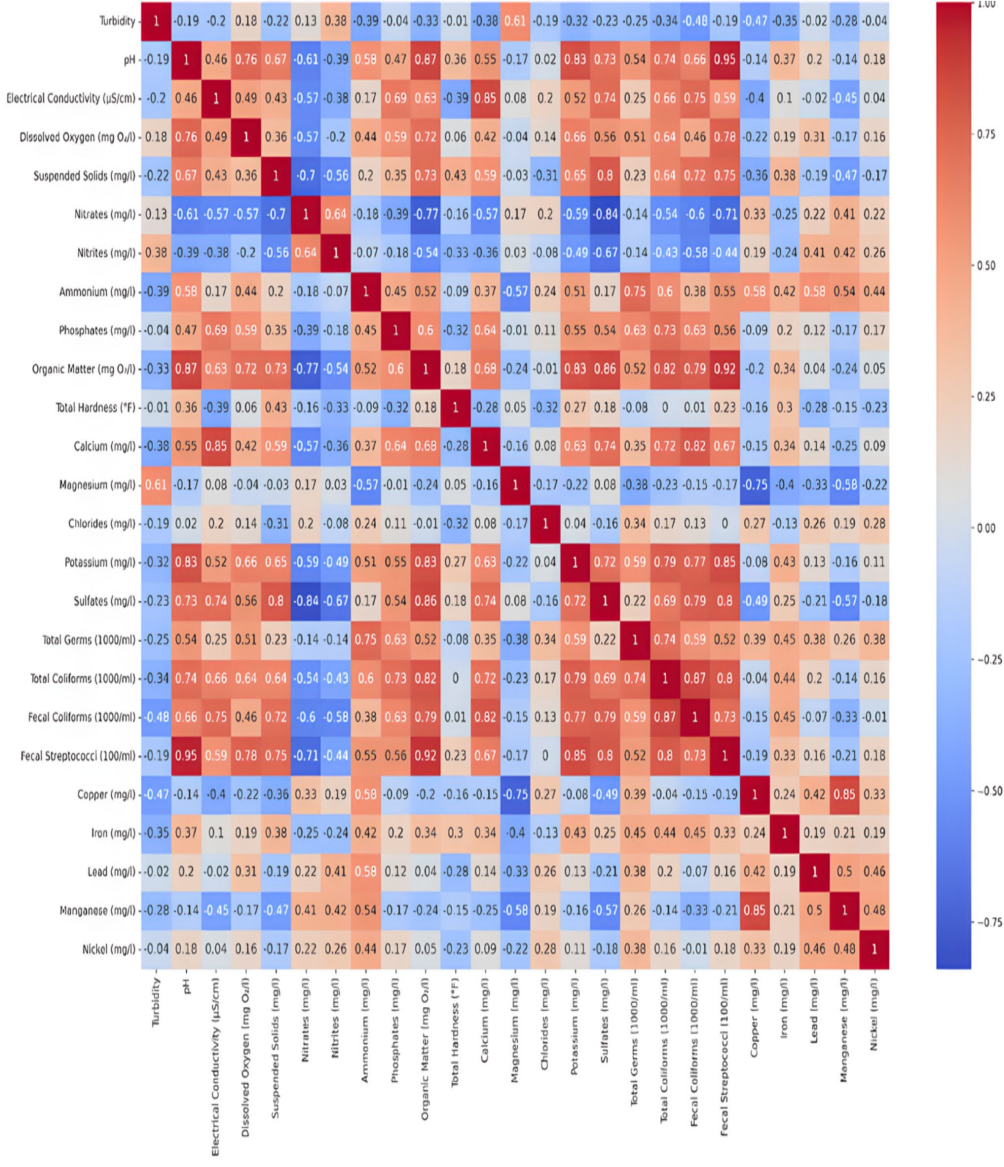
Non-polluted wetlands: correlation analysis of physicochemical and microbiological parameters

The correlation matrix in non-polluted wetlands highlights key relationships among water quality parameters. Diagonal values of 1.0 indicate perfect self-correlation, while off-diagonal values reveal interactions between variables. Turbidity shows a weak negative correlation with pH (r = − 0.19), suggesting slight pH decreases correspond with small increases in turbidity. Electrical conductivity strongly correlates with calcium (r = 0.85), reflecting calcium’s influence on conductivity. Organic matter is strongly associated with fecal streptococci (r = 0.92) and pH (r = 0.87), indicating high organic content promotes microbial growth and elevated pH. Nitrates negatively correlate with organic matter (r = − 0.77), likely due to nitrate-reducing bacterial activity. Moderate correlations, such as between turbidity and suspended solids (r = 0.47), further highlight parameter interdependence (Fig. 6).

#### Regional wetland analysis

**Fig. 7:**
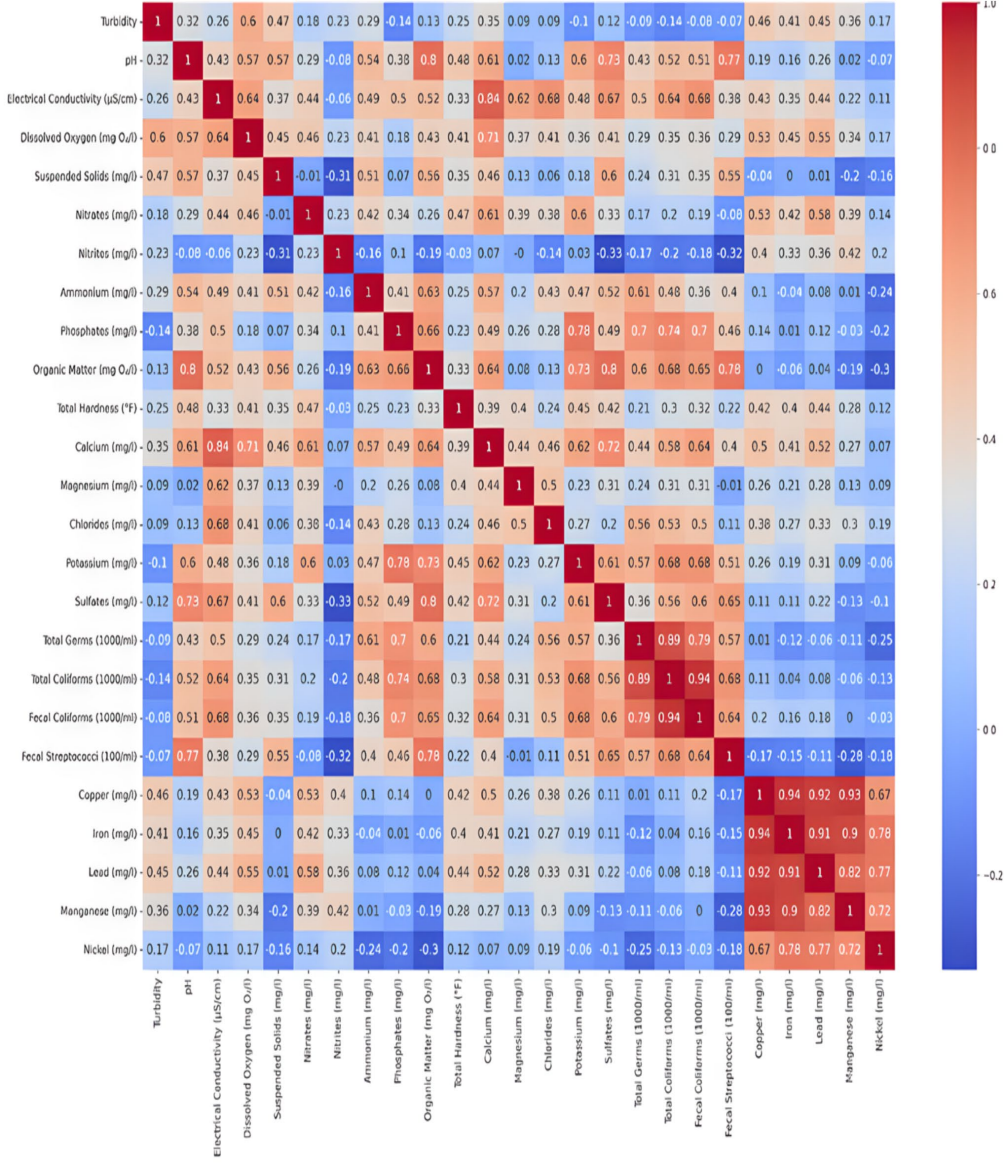
All region wetlands: Correlation analysis of physicochemical and microbiological parameters

The correlation matrix highlights complex relationships among wetland water quality parameters. Diagonal values are 1.0, while off-diagonal values reveal key interactions. Turbidity and pH are weakly negatively correlated (r = − 0.32), whereas electrical conductivity strongly correlates with calcium (r = 0.84). Significant positive correlations include organic matter with sulfates (r = 0.8) and pH with organic matter (r = 0.8). Moderate correlations exist between turbidity and suspended solids (r = 0.47) and ammonium and phosphates (r = 0.63). Total germs and total coliforms show a very strong correlation (r = 0.89), emphasizing coliforms as key indicators of microbial contamination (Fig. 7).

**Fig. 8:**
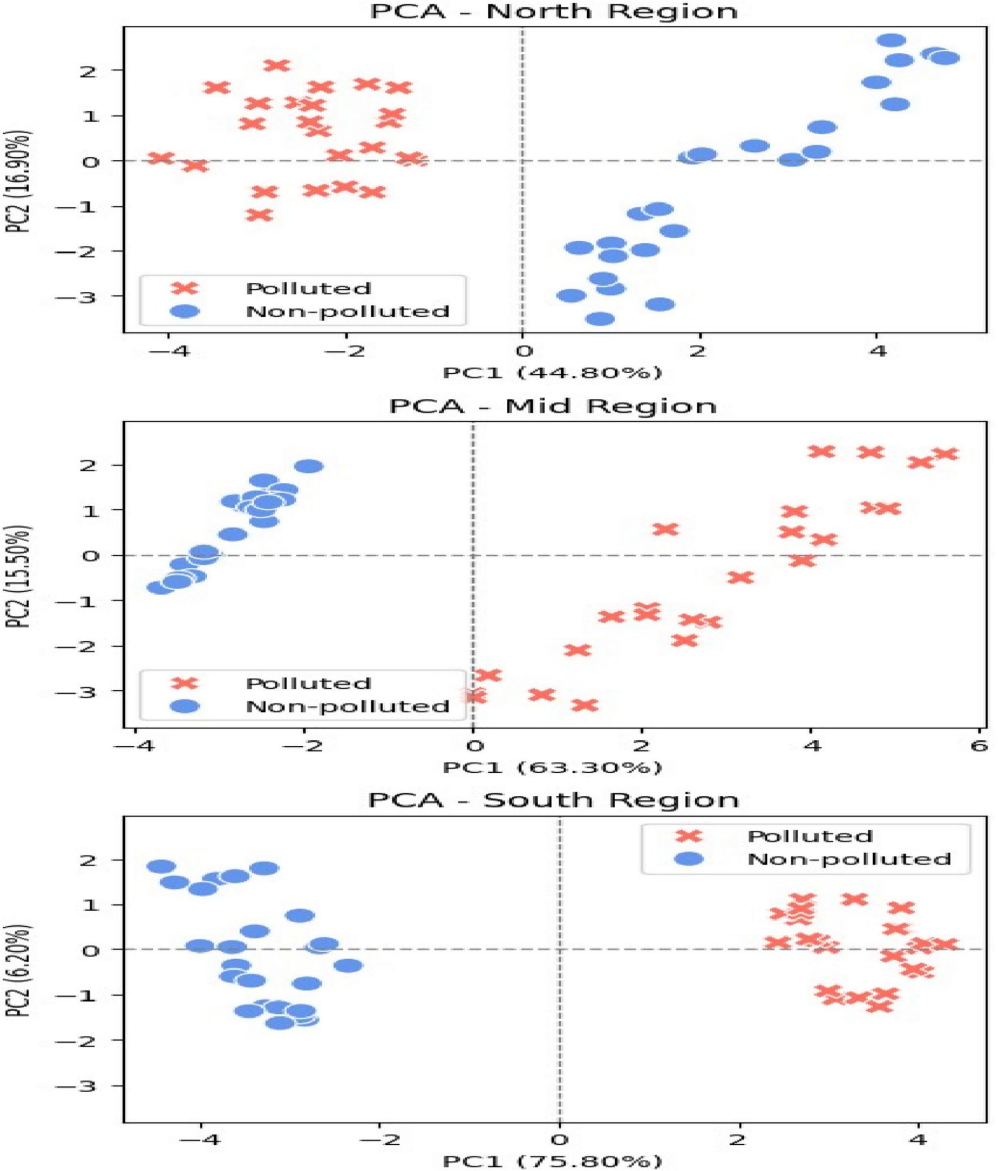
Principal component analysis (PCA) of water quality in North, Mid, and South regions

We used a refined subset of 15 important physicochemical and microbiological parameters in a principal component analysis (PCA) to reduce dimensionality and clarify the underlying structure of our water quality data. Only parameters with factor loadings and communalities ≥ 0.4 were kept after these were chosen for their contribution to the principal components. We used Bartlett’s Test of Sphericity and the Kaiser–Meyer–Olkin (KMO) Test to make sure our dataset was appropriate before PCA. The findings validated the suitability of the data structure for PCA (KMO > 0.8 for the global dataset and > 0.7 for the majority of regional subsets; Bartlett’s *p* < 0.001). The main environmental gradients and pollution impacts throughout the study regions were succinctly and clearly summarized by the PCA results. The global dataset was analyzed to identify a consistent set of variables, which were then applied consistently throughout all regions to allow for comparison. The data from each geographic region (northern, highland, and southern wetlands) was subjected to separate principal component analysis (PCA) in order to look for patterns in the multivariate dataset and reduce dimensionality while maintaining the maximum variability (Fig. 8). The first two principal components, PC1 and PC2, which offered a two-dimensional depiction of the data structure, served as the foundation for the analysis. Regional differences existed in the cumulative variance explained by PC1 and PC2. The cumulative variance in the northern wetlands was 61.7%, with PC1 explaining 44.8% of the total variance and PC2 explaining an additional 16.9%. PC1 and PC2 each contributed 63.3% and 15.5% to the explanation of the highland (mid) wetlands, for a total of 78.8%. The cumulative variance in the southern wetlands was 82.0%, with PC1 accounting for 75.8%of the variability and PC2 contributing 6.2%. These results suggest a larger dimensional decrease in the south, suggesting that a dominant underlying gradient (perhaps related to environmental or pollution-related factors) is responsible for the variability observed. The northern variation is, on the other hand, more uniformly distributed across multiple components, indicating more complexity or heterogeneity in the factors that affect the northern region. Sampling adequacy and suitability of the data for PCA were evaluated using the Kaiser–Meyer–Olkin (KMO) statistic and Bartlett’s test of sphericity. The overall dataset exhibited excellent sampling adequacy (KMO = 0.84), and Bartlett’s test confirmed significant correlations among variables (χ^2^ = 3806, *p* < 0.001). Regional subsets showed similar factorability: the South (KMO = 0.86, χ^2^ = 1653, *p* < 0.001) and the Mid region (KMO = 0.76, χ^2^ = 1874, *p* < 0.001) demonstrated strong sampling adequacy, while the North region presented a lower yet acceptable value (KMO = 0.55, χ^2^ = 1370, *p* < 0.001). All *p* values were < 0.001, indicating that the correlation matrices were appropriate for PCA extraction.

## Discussion

This study conducts a comprehensive assessment of water quality and pollution level in the wetlands of Algeria. It highlights notable variations in physicochemical properties, microbiological content, and heavy metal concentrations. These differences are largely influenced by geographic factors, which play a crucial role in determining the overall health of these vital ecosystems and their susceptibility to environmental challenges.

One of the pivotal findings of the research is the notable increase in turbidity and suspended solids observed in polluted wetland areas, particularly those situated along the coastal and desert regions. This observation aligns with previous studies that have established a clear link between heightened turbidity levels and the influx of urban and industrial runoff. Such runoff contributes significantly to the degradation of water quality, as it carries a cocktail of pollutants that disrupts the natural balance of these ecosystems. (Müller et al. 2020; Taylor & Owens 2009; Q. Wang et al. 2017).

Increased suspended solids concentrations and elevated turbidity have been reported in several Algerian wetland areas, suggesting high pollution levels that affect aquatic ecosystems and water quality (Fashagba et al. 2024; Fekrache and Boudeffa 2019; Karim et al. 2024). Increased turbidity in aquatic environments significantly disrupts photosynthesis, hindering plant growth and oxygen production. This murkiness also alters the behaviors of animals, affecting their feeding and mating patterns, while influencing predator–prey dynamics within the ecosystem (Figueiredo et al. 2016; Gutierrez et al. 2021; Wang et al. 2022). Elevated levels of nitrates, ammonium, and phosphates were found in highland wetlands, highlighting nutrient enrichment. Agricultural runoff from the area’s intensive farming methods is primarily to blame for this phenomenon. When fertilizers are used excessively in agriculture, nutrients leak into surrounding water bodies, causing eutrophication, a condition that can negatively affect aquatic life (Withers et al. 2014). An additional study indicates that nitrates, ammonium, and phosphates are prevalent in the Esbikha wetlands of northeastern Algeria. The high levels of nitrogenous compounds (up to 124 mg/L of NO_3_) and orthophosphates (0.2 mg/L of PO_4_) degrade water quality, exceeding the World Health Organization’s (WHO) recommendations (Mamour et al. 2024). The elevated nitrate and phosphate levels observed in the Boussedra and Sebkhet Bazer-Sakra wetlands align with findings from Lake Taihu in China, where agricultural runoff and domestic effluent contributed to eutrophication and algal blooms (Qin et al. 2007, 2019). Similarly, the Chott Oum Raneb site’s high levels of microbial contamination are similar to those found in wetland systems in Bangladesh and Uganda, where livestock activity and sanitation were significant factors (Ahumuza 2014; Hossain 2015; Kansiime and Maimuna 1999). There has been significant contamination of the Mitidja alluvial aquifer, a major source of drinking, industrial, and agricultural water. Among 1185 nitrate measurements taken at 316 sampling points between June 1985 and May 2015, 423 samples exceeded the 50 mg/L nitrate consumption limit at 84 observation points. There was particularly severe contamination in the eastern and western aquifer watersheds (Lagoun et al. 2021a, b). A study identified elevated levels of nitrate because of hydrogeological factors (including the aquifer characteristics, the depth of the water table, and the thickness of the saturated zone) as well as human activities (such as land use, agricultural practices, and population density) (Lagoun et al. 2021a, b). Although precise information on the levels of nitrite in the lakes, rivers, and wetlands of northern Algeria is limited, it is crucial to remember that nitrite is an unstable form of nitrogen found in natural waters and is usually found in smaller amounts than nitrate. However, both nitrate and nitrite are bioavailable forms of nitrogen that can cause eutrophication, which degrades water quality and damages aquatic ecosystems by causing an overabundance of algae and aquatic plants (EPA 2021). Organic pollution reduces dissolved oxygen (DO) levels which negatively affects wetland functioning such as decomposition and nutrient cycling. The lowering of DO levels fosters anaerobic conditions, resulting in increased accumulation of dissolved organic carbon (DOC) due to increased activity of hydrolase enzymes and decreased conversion of hydrolysates to gaseous carbon (Chen et al. 2011).

Increasing phosphorus concentration and its impact on dissolved oxygen balances in wetlands like Everglades in subtropical Florida (McCormick and Laing 2003) facilitate the drop of DO levels, which can increase further anaerobic situation that impacts carbon cycling and denitrification in organic pollution impacted wetlands. For instance, the organic contamination of the Churni River in India is predominantly due to the dumping of industrial wastewater. As a result of the action of bacteria in organic waste decomposition, the level of DO has drastically decreased to 0.2 mg/L and the Biochemical Oxygen Demand BOD is 68 mg/L. This dilapidating oxygen deficiency has resulted in reduced habitat quality, odd physiological responses in fishes, and reduction in fish population (Das et al. 2020). In all three polluted regions, environmental thresholds were observed to be exceeded, indicating the extent of human influence on Algerian wetlands. High levels of phosphate and nitrogenous compounds are a sign of agricultural runoff and untreated wastewater discharge, which are frequently linked to algal blooms and eutrophication (Bouchaala et al. 2025; Wang et al. 2019). Significant risks to public health, especially in rural areas that depend on wetland water, are presented by high levels of fecal indicators like coliforms and streptococci, which indicate contamination from domestic or livestock waste. The danger of bioaccumulation in aquatic organisms is further highlighted by the discovery of heavy metals like lead and nickel that are above WHO and Algerian standards. Both natural aridity and potential industrial saline discharge are reflected in elevated electrical conductivity values, particularly in highland and desert locations. Strong wastewater treatment infrastructure, effluent standard enforcement, and the implementation of routine monitoring programs in accordance with international standards are all necessary, as these findings highlight (EPA 2023; WHO 2008).

Alongside this, the United States Environmental Protection Agency (US EPA) claims that in pristine, natural flowing streams, the DO concentrations are usually adequate to support robust ecosystem. Moreover, some fish and invertebrates may be harmed. The presence of microbial indicators such as fecal coliforms and fecal streptococci across several sites, particularly in Boussedra Wetland (coastal region) and Chott Oum Raneb (southern region), raises significant public and ecological health concerns. These markers are frequently used to evaluate the presence of pathogenic organisms in aquatic environments and fecal contamination (Cabral 2010). According to the World Health Organization (WHO 2008, 2017, 2018), fecal coliform concentrations exceeding 1000 CFU/100 ml in recreational waters indicate a high risk for waterborne disease transmission, while concentrations above 100 CFU/100 ml render water unsuitable for unrestricted irrigation. We found that values from polluted sites exceeded these values, suggesting contamination from untreated sewage, agricultural runoff, or livestock in nearby catchments. Higher microbial loads also pose risks of gastrointestinal infections, hepatitis A, and parasitic diseases in exposed human populations, as well as gastrointestinal infections, hepatitis A, and parasitic diseases in exposed human populations (Ashbolt 2004), but also degrade wetland ecosystem health by increasing nutrient loads, promoting eutrophication, and reducing dissolved oxygen availability or even die due to low or very high DO levels that are often the result of organic pollution (Wotton 1995). In polluted wetlands, the presence of dangerous microorganisms like fecal streptococci and coliforms is a major concern because these bacteria are markers of fecal contamination and possible health hazards. Water quality is frequently evaluated using fecal streptococci and coliforms, including *Escherichia coli*. Their existence suggests that warm-blooded animals, including humans, have contaminated the feces (US EPA 2012). Research has demonstrated that compared to other indicator organisms, fecal streptococci are typically more resilient to environmental factors and purification procedures. They are trustworthy markers of fecal pollution because they frequently last longer in natural water environments (Cohen and Shuval 1973). The microbiological evaluation includes fecal coliform, total coliform, actinomycetes, fungi, and bacteria. The presence of fecal coliform indicates that the contaminated fecal material is an indirect source of organic matter that has broken down (Fadhil Abo-Ksour et al. 2017; Kumar et al. 2018). Fecal pollution, which endangers both human and animal health, is indicated by the presence of fecal streptococci and coliforms in water bodies. To evaluate water quality and guarantee the security of aquatic environments, it is imperative to monitor the changes of these indicators (Some et al. 2021). The elevated concentrations of heavy metals in contaminated wetlands pose a significant long-term risk to ecosystems and food chains. These dangerous substances can affect human health because they can linger in the environment, cause organisms to bioaccumulate, and biomagnify through trophic levels. Arsenic (As), cadmium (Cd), mercury (Hg), and lead (Pb) are some of these elements (Shome et al. 2025). Heavy metals are often sucked up by wetland sediments. The geochemical cycling of these metals can be altered by environmental changes, such as changes in water levels, and may result in their reintroduction into the water column. This remobilization may increase ecological risks that affect wildlife and human populations (Yin et al. 2020). Numerous investigations have revealed this kind of contamination in wetlands all over the world. The pervasive problem of heavy metal pollution in coastal wetlands was brought to light by a thorough review that examined 3343 studies published between 1990 and 2019. The study underlined the necessity of conducting methodical evaluations to comprehend the scope and consequences of such contamination (Li et al. 2022). Another study stated that numerous sources of heavy metal pollution were found during investigations in the sediments of the Anzali Wetland, Iran (Zarei et al. 2023). Research on the Ashtamudi Wetland in India has shown that industrial and urban runoff significantly contaminates the environment with heavy metals. Seasonal fluctuations in contamination levels were noted, with sometimes exhibiting high levels of metals such as lead and cadmium, which could be harmful to the wetland’s ecology (Anjana et al. 2025). According to reports, landfill operations have caused serious pollution in the San Silvestre wetlands in Colombia. Poor water quality, widespread fish kills, and negative health impacts on nearby communities have all resulted from the contamination (Aib et al. 2025; Iñigo Alexander 2025). Studies conducted in Algeria’s Guenitra Basin have shown that the dam reservoir has high turbidity levels, up to 50 NTU. This increase emphasizes the effect of human activity on water quality and is ascribed to soil erosion, surface runoff, and the inflow of suspended solids from household waste sources (Fekrache and Boudeffa 2019). The non-biodegradable nature of heavy metals and their detrimental effects on biota make their persistence and toxicity in wetland ecosystems concerned. Water and soil quality can deteriorate due to contamination, which can have a negative impact on plant and animal life (Yaşar Korkanç et al. 2024). Effective management techniques, such as pollution prevention, remediation, and restoration initiatives, are necessary to address heavy metal contamination in wetlands in order to minimize negative effects and maintain ecological integrity (El-Sharkawy et al. 2025). Some water quality parameters in different regions vary (or even lack) according to geological, hydrological, and anthropogenic factors. According to geochemical studies demonstrating stable ionic concentrations in mineral-rich wetland sediments, the comparatively constant magnesium concentrations across both polluted and reference sites most likely reflect geological background levels and groundwater-surface water interactions. According to geochemical studies demonstrating stable ionic concentrations in mineral-rich wetland sediments, the comparatively constant magnesium concentrations across both polluted and reference sites most likely reflect geological background levels and groundwater-surface water interactions (Bini et al. 2011; Gonçalves et al. 2022; Gustafson and Wang 2002; Kelepertsis et al. 2001; Kirsch 2020; Nilsson et al. 2023; Stanton et al. 2007). On the other hand, high nitrate and phosphate levels are consistent with results from agricultural catchments, where it has been shown that land-use intensity greatly raises nutrient loads (Gustafson and Wang 2002), for example: 0.62–1.35 mg/L NO₃⁻, 0.07–0.37 mg/L TP) compared to forested regions. Furthermore, disparities in the retention efficiencies of nitrogen and phosphorus in prairie-pothole wetlands highlight the crucial roles that substrate, hydrology, and ecoengineering play in forming nutrient profiles; these differences are instructive for interpreting variability in the mid- and southern regions (USDA 2023). Exceedances of ammonium and phosphate thresholds in both polluted and non-polluted sites highlight nutrient enrichment as a widespread issue, likely linked to agricultural runoff and untreated domestic wastewater. Similarly, the near-universal non-compliance of microbial indicators underscores sanitation gaps and the potential public health risk for communities depending on these wetlands. In contrast, most metal concentrations (e.g., iron, copper, nickel) remained within international limits, with the exception of lead, which exceeded thresholds in Boussedra and Sebkhet Bazer-Sakra. These patterns reflect the combined influence of land use, wastewater inputs, and hydrological variability across regions.

Hydrological characteristics strongly influence pollutant distribution and seasonal variability in wetlands, yet these factors differ substantially among the Algerian systems studied. Wetlands with lower flow velocity and weak hydrological flushing tended to exhibit higher concentrations of nutrients (NO₃⁻, NH₄⁺, PO₄^3^⁻) and suspended solids, consistent with global findings showing that reduced water exchange promotes nutrient retention and microbial proliferation (Burns 2005; Whigham et al. 1988). Similarly, shallow wetlands with limited vertical mixing—such as those in the northern region—showed greater temporal fluctuations in temperature and dissolved oxygen, reflecting the sensitivity of shallow systems to heat pulses and biochemical oxygen demand (Bird et al. 2025). Connectivity also played a role: wetlands receiving continuous inflow from agricultural drains or seasonal wadis showed distinct pollutant signatures compared to more isolated basins, supporting recent global evidence that hydrological connectivity governs pollutant transport and dilution capacity (Global Wetland Outlook 2025; Tockner et al. 1999). Overall, the observed spatial gradients in Algerian wetlands align with international research indicating that flow regime, depth, residence time, and lateral connectivity are critical drivers of water-quality patterns in wetland ecosystems.

Regional climatic conditions had a significant influence on the seasonal and spatial variations in water quality parameters found throughout the study wetlands. The increased turbidity and fecal indicator loads observed during the wet season in coastal wetlands are explained by high winter rainfall, which also increases surface runoff and speeds up the movement of suspended solids, nutrients, and microbial contaminants into receiving (IPCC 2014). In contrast, cooler temperatures and moderate rainfall in the highland region affect the accumulation of nutrients, especially nitrates and ammonium, which are released from agricultural fields during precipitation events (Giri and Qiu 2016). The strong evaporative demand typical of arid climates, which raises solute concentration and decreases dilution capacity, is consistent with the highest concentrations of dissolved salts, suspended solids, and heavy metals found in desert wetlands (Kalff 2002). These climatic effects are consistent with global evidence demonstrating that temperature, rainfall variability, and evaporation rates are primary drivers of physicochemical and microbiological dynamics in Mediterranean, semi-arid, and arid wetland ecosystems (Ferreira et al. 2024).

## Conclusion

This study provides a comprehensive assessment of water quality in various wetlands across Algeria, focusing on the detrimental effects of pollution on key physicochemical, microbiological, and heavy metal parameters. By conducting a comparative analysis of coastal, highland, and desert wetlands, the research uncovers distinct pollution patterns that are shaped by a combination of geographic, climatic, and anthropogenic factors.

The findings reveal a concerning trend: polluted sites exhibit elevated levels of turbidity, electrical conductivity, nutrient concentrations, heavy metal content, and microbial contamination. In stark contrast, non-polluted areas demonstrate significantly better ecological health and stability. Notably, the study identifies significant regional disparities in pollution sources and impacts. Coastal wetlands, situated near urban and industrial centers, face severe degradation due to industrial discharges and urban runoff. In contrast, highland wetlands are primarily affected by agricultural runoff, which introduces excess nutrients and chemicals into these sensitive ecosystems. Desert wetlands, while more remote, are not exempt from pollution; they experience extreme turbidity largely attributable to the harsh arid conditions that exacerbate sediment disturbance. The comparison with international and national standards provides quantitative evidence of the severity of pollution in Algerian wetlands, allowing a transparent distinction between polluted and non-polluted sites. Algerian wetlands experience different seasonal pressures, as shown by the comparison of the wet and dry seasons (Fig. 4). The wet season is characterized by microbial contamination, while the dry season is dominated by nutrient enrichment. In order to effectively manage, seasonal dynamics should be incorporated, with a focus on microbial pollution mitigation during rainy seasons and nutrient control during droughts.

This research underscores the urgent need for targeted conservation efforts to protect these vital ecosystems from further degradation. By implementing tailored management strategies that address the unique challenges faced by each wetland type, stakeholders can work toward restoring ecological balance and enhancing water quality. Ultimately, the study advocates for increased awareness and action to mitigate pollution and safeguard the diverse biological and ecological functions of Algeria’s wetlands. In addition to conservation strategies, we recommend several concrete management actions: the establishment of riparian buffer strips to reduce nutrient runoff; strengthening of wastewater treatment facilities in polluted wetlands to reduce untreated discharges; development of long-term monitoring programs aligned with WHO and EPA guidelines; and promotion of community awareness and education campaigns in agricultural zones to encourage sustainable practices. These measures are particularly important in areas where untreated effluent and agricultural runoff are causing water quality degradation.

We advise the immediate establishment of vegetative buffer zones around susceptible wetlands, more stringent enforcement of wastewater discharge laws, and routine water quality monitoring in accordance with international standards in order to promote successful wetland conservation. These measures are particularly important in areas where untreated effluent and agricultural runoff are causing water quality degradation. Our results provide a scientific basis for determining wetland management strategies in Algeria and similar arid and semi-arid regions, and contribute to national and international efforts to preserve and maintain wetland.

### Practical management recommendations for Algerian wetlands

1. Establish a national wetland water-quality monitoring network.

The Ministry of Environment and the National Agency for Nature Conservation (ANN) should standardize wetland monitoring across the northern, highland, and southern regions by implementing unified sampling protocols, seasonal sampling schedules, and a centralized data repository.

2. Integrate hydrological data (flow, depth, residence time) into routine assessments.

Wetlands with weak flushing (e.g., Mellah, Reghaia) require priority management due to higher pollutant retention. Installing basic hydrometric sensors would allow authorities to track water residence time and predict contamination risk.

3. Regulate agricultural nutrient inputs in buffer zones.

Based on the observed elevated NO₃⁻, NH₄⁺, and PO₄^3^⁻ loads, the Ministry of Agriculture should enforce 100–300 m vegetated buffer strips around agricultural wetlands, especially in the northern region. Agrochemical application schedules should avoid peak runoff periods (January–March).

4. Strengthen wastewater pre-treatment for communities surrounding wetlands.

Many Algerian wetlands receive untreated or partially treated municipal effluents. Municipal water agencies should upgrade pre-treatment systems near wetland inflows, prioritizing regions where COD, BOD₅, or microbial contamination exceeded WHO/FAO limits.

5. Implement heavy-metal source tracing and control measures.

For wetlands in industrial zones (particularly highland sites), targeted inspections of small industries, workshops, and slaughterhouses should be conducted to trace Cu, Fe, and Pb discharges. Mandatory sediment traps or constructed wetlands can be required when hotspots are identified.

6. Develop ecological buffer belts for high-risk wetlands.

Wetlands with strong anthropogenic pressure (e.g., agricultural runoffs in the north, livestock grazing in highlands) should be protected by ecological buffer belts consisting of native reed beds (Phragmites australis) and halophytes that naturally filter nutrients and metals.

7. Promote community-based wetland stewardship programs.

Local associations should be involved in monitoring illegal dumping, managing grazing intensity, and reporting wastewater leakage—practices already successful in the Mediterranean region.

8. Integrate wetland management into national climate adaptation plans

Given the strong seasonal fluctuations observed in southern wetlands, the National Climate Adaptation Strategy should incorporate wetland hydrological resilience (e.g., maintaining connectivity during droughts) as a priority action.

## Data Availability

The corresponding author can provide the datasets created and examined during the current study upon reasonable request.
